# Editorial Notes

**Published:** 1928

**Authors:** 


### Editorial Notes.

**The King's Health.**

The anxiety caused by the King's serious illness has to some extent been relieved. The facts about his illness which have been recently published prove how well-grounded that anxiety has been. A streptococcal septicæmia, accompanied by pulmonary consolidation and empyema, is a malady from which recovery is barely to be hoped for. There is, indeed, cause for thankfulness that His Majesty seems to have passed safely through a phase in which he fell into a typhoid state with periods of grave myocardial failure. As we go to press the bulletins report his continued though slow progress towards what we all hope will be complete restoration to health. It is in times of sickness that a nation can manifest by its solicitude and sympathy the loyalty and gratitude which King George V. has richly earned from his subjects throughout the Empire. To him in his suffering as well as to the Queen and the Royal Family in their anxious waiting and watching the deepest sympathy is due. A word of grateful appreciation may be expressed also to the King's doctors for their wise care of their royal patient, and for the clear and full information which they have published as to the nature and progress of his illness. The public has been admitted to a degree of confidence which is as admirable as it is uncommon when a Sovereign's life is in jeopardy.

**Hospital Ball for the Lord Mayor's Hospital Fund.**

As part of the University effort on behalf of the Lord Mayor's collection for the Bristol hospitals a most successful Ball was held at the Victoria Rooms on Wednesday, January 2nd, 1929. The attendance reached the satisfactory number of 200. The Lord Mayor accompanied by the Lady Mayoress came and made a speech, in which he paid graceful compliments to the University Union and the Committee of ladies who had organised the Ball. His speech was short and appropriate, and the length of his stay was a compliment no less marked. In connection with the special efforts which the University makes year by year to swell the Lord Mayor's Hospital Fund, it may not be out of place to suggest that the expression "University Rag" should be discontinued. A rag is defined in the dictionary as "noisy, disorderly conduct." Hitherto the conduct of the students who have organised the University collections for the Lord Mayor's Fund has not deserved such a description. The "Press" is not wholly free from blame in this matter. The students' collection is given publicity and free advertisement under the title of "The University Rag." That the affair has been carried out with a maximum of efficiency and fun and a minimum of horseplay and rowdiness is attributable to the good manners and breeding of the students rather than to any encouragement they may derive from the inappropriate headlines under which the newspapers report their doings. The University students work very hard for the money they collect. If they amuse the public more than they amuse themselves the public charities are the gainers. But to call this "ragging" is surely a derangement of epithets.

